# Sex and vision II: color appearance of monochromatic lights

**DOI:** 10.1186/2042-6410-3-21

**Published:** 2012-09-04

**Authors:** Israel Abramov, James Gordon, Olga Feldman, Alla Chavarga

**Affiliations:** 1 Psychology, Brooklyn College, City University of New York, Brooklyn, NY, 11210, USA; 2 Cognition, Brain, and Behavior, The Graduate Center, City University of New York, New York, NY, 10016, USA; 3 Psychology, Hunter College, City University of New York, New York, NY, 10065, USA; 4 Biopsychology and Behavioral Neuroscience, The Graduate Center, City University of New York, New York, NY, 10016, USA; 5 Center for Neural Science, New York University, New York, NY, 10003, USA

## Abstract

**Background:**

Because cerebral cortex has a very large number of testosterone receptors, we examined the possible sex differences in color appearance of monochromatic lights across the visible spectrum. There is a history of men and women perceiving color differently. However, all of these studies deal with higher cognitive functions which may be culture-biased. We study basic visual functions, such as color appearance, without reference to any objects. We present here a detailed analysis of sex differences in primary chromatic sensations.

**Methods:**

We tested large groups of young adults with normal vision, including spatial and temporal resolution, and stereopsis. Based on standard color-screening and anomaloscope data, we excluded all color-deficient observers. Stimuli were equi-luminant monochromatic lights across the spectrum. They were foveally-viewed flashes presented against a dark background. The elicited sensations were measured using magnitude estimation of hue and saturation. When the only permitted hue terms are red (R) yellow (Y), green (G), blue (B), alone or in combination, such hue descriptions are language-independent and the hue and saturation values can be used to derive a wide range of color-discrimination functions.

**Results:**

There were relatively small but clear and significant, differences between males and females in the hue sensations elicited by almost the entire spectrum. Generally, males required a slightly longer wavelength to experience the same hue as did females. The spectral loci of the unique hues are not correlated with anomaloscope matches; these matches are directly determined by the spectral sensitivities of L- and M-cones (genes for these cones are on the X-chromosomes). Nor are there correlations between loci of pairs of unique hues (R, Y, G, B). Wavelength-discrimination functions derived from the scaling data show that males have a broader range of poorer discrimination in the middle of the spectrum. The precise values for all the data depend on whether Newtonian or Maxwellian optics were used, but the sex differences were the same for both optical systems.

**Conclusion:**

As with our associated paper on spatio-temporal vision, there are marked sex differences in color vision. The color-appearances we measured are determined by inputs from thalamic neurons (LGN) to individual neurons in primary visual cortex. This convergence from LGN to cortex is guided by the cortex during embryogenesis. We hypothesize that testosterone plays a major role, somehow leading to different connectivities for males and females: color appearance requires a re-combination and re-weighting of neuronal inputs from the LGN to the cortex, which, as we show, depends on the sex of the participant.

## Background

We are studying the ways in which the visual system processes the image that is focused onto the retina at the back of the eyeball. In the companion paper to this one, we examined the ways by which vision resolves spatial and temporal variations in stimuli – that is, changes in light and dark across the image; we found significant differences between males and females [1].

In this paper we report on sex differences in color vision. There are several reasons why it is especially interesting to study color vision: color vision may well have the longest history of detailed studies of sensory mechanisms, which means that we have a large background on which to build. Furthermore, we now have an excellent understanding of the genetic bases for the initial steps by which light is converted into a neuronal signal. And some of these bases are sex-linked: color vision depends on three types of cones, some of which are more sensitive to the longer wavelengths of light (L-cones), some to the middle wavelengths (M-cones), and some to the shorter wavelengths (S-cones). The genes coding for two of these cone photoreceptors (L- and M-cones) are carried on the X-chromosome.

Sex differences have been noted for various basic sensory functions. For example, in the auditory system females have better hearing sensitivity than males; these and other differences can be related directly to the masculinizing effects of androgens [2-4]. For the olfactory system, a recent, large review of the literature concluded that, in most cases females had better sensitivity, and discriminated and categorized odors better than males [5]. At least for these sensory modalities, and also for taste and somato-sensory sensitivity, females do better than males [6].

Gonadal steroid hormones may be the basis for these sex differences. In rhesus monkeys, many androgen receptors are found on neurons throughout the cerebral cortex, including visual cortex [7]. There are similar findings for rats, in whom males have more androgen receptors than females, and these are especially plentiful in primary visual cortex [8]. A recent review has reiterated these findings and concluded that in both humans and rats the largest concentration of androgen receptors in the forebrain is in the cerebral cortex and not the hypothalamic and limbic areas associated with reproduction [9]: these findings would seem to be general across mammals.

Furthermore, in rats, it is the androgens, and not estrogen, that directly affect development of the visual cortex. Early post-natal cell-death (apoptosis) of the visual cortex is reduced by androgens; as a result males have 20% more neurons in the visual cortex [10,11]. This organizational effect is androgen-specific: early exposure of female rats to androgens (implanted capsules of dihydrotestosterone) led to these effects; early exposure to estrogen (implanted capsules of estradiol) did not inhibit post-natal cell-death [11]. Because the genes for the L- and M-cones are on the X-chromosome, females might have a double "dose" of sex-related genes. To compensate for this, one of each pair of X-chromosomes is silenced [12]. Furthermore many humans have multiple L- and M-genes – we are polymorphic for these genes [13,14]. And different retinal areas might express different alleles, which would affect the responses of these areas and the brain sites associated with different retinal areas. Moreover, the X-chromosome may have a loading of "male-benefit" genes: thus, any recessive alleles must, of necessity, be expressed in a male [15]. Furthermore, some of the sex effects we find could be either organizational or activational and could depend on estrogen rather than testosterone; they could even be due to other sex-related genes [16].

Speculatively, however, the preponderance of testosterone receptors in male brains may be the basis for differences in thalamo-cortical connections: early in development axonal growth towards the cortex is in part guided by projections from cortex to the thalamus [17]; and these could be affected by variations in gonadal hormones.

Very few studies of color vision, other than those dealing directly with L- and M-cone genes, look for sex differences. Our focus here is particularly on color appearance. We are not considering, therefore, studies of color vision with cognitive or culture-bound effects: for example, reports that among English speakers, women have a larger vocabulary for describing color stimuli than do men [18,19]; also, some cross-cultural studies show that women's color preferences are not the same as those of men [20].

Color sensations can be described along three separate dimensions: hue, saturation, and brightness. Hue is what is commonly referred to as "color" – red, or yellow, or green. Saturation is how deeply colored is the sensation – compare fire-engine red with a pastel red (pink) – the former is highly saturated, while the latter is less saturated; and white is totally desaturated. Brightness has its ordinary everyday meaning -- stimuli ranging from black through grays to white vary in brightness.

A few of the small number of studies that have dealt directly with color appearance used colored samples (Munsell standard reflectance chips). In one study, a form of Multidimensional Scaling (MDS) was used to find similarities among a set of Munsell stimuli and to derive a form of color space [21]. But with these sorts of reflectance stimuli, it is not possible to get a wide range of hues of high saturation, while keeping all at approximately the same brightness. (In Munsell terms, this would mean creating chips of high chroma and high value; for reflecting objects seen on a background, the correct term for "brightness" is "lightness.") Because of the problems with separating the dimensions of these stimuli, the investigators had to place various restrictions on the possible solutions from MDS. The major conclusions were that males placed less weight on inter-stimulus separation along a red-green axis but more on a lightness axis as compared to females. However, as the authors admit, the findings may reflect sex differences in cultural factors relating to range of available color terms and access to them.

As part of a battery of visual tests that we have been applying uniformly for some years to large samples of participants, we use magnitude estimation techniques to measure hue and saturation of flashes of monochromatic lights; the intensities of all of these stimuli were adjusted to make them equal in luminance (approximately equal in "brightness"). Our magnitude scaling methods, derived directly from Hurvich and Jameson [22], require participants to assign numbers to the sensations elicited by each stimulus. To do this, we use a strict protocol (described below), whose reliability and validity we have explored quite extensively [23-27]. We used two optical systems: about half the participants viewed the stimuli with their natural pupils (Newtonian-View); for the others, the light from a second optical system was focused through the central 2 mm zone of the pupil (Maxwellian-View); in both cases, the illuminance on the retina was the same.

Our magnitude scaling technique uses a continuous scale to describe the hue and saturation of stimuli. It should be noted that this is fundamentally different from hue-naming in which continuous curves are obtained mostly because participants are not entirely consistent in the names they use from trial to trial. Participants find our magnitude estimation procedure easy, it is highly reliable and rapid -- a complete data set, with all repeats, requires less than one hour. Also, from one set of data we can derive a variety of other functions, such as wavelength discrimination, with the same precision as if that function was the only one being measured [27].

The method is very simple: we ask participants to describe their sensations, but in a highly controlled fashion. The necessary and sufficient terms needed to describe hue completely are Red (R), Yellow (Y), Green (G), and (B) [28]; a complete description also needs a term for saturation. Unlike most linguistic terms, the basic color terms have universal denotations [29], and therefore can be used to inform us about functions common to the entire species rather than to the vagaries of a particular group’s language (see [30] for a review). Of course, a participant's native language must have lexical equivalents for R, Y, G, and B, otherwise they could not perform our task [24,31].

A term for "brightness" is not needed in our studies because all our stimuli are equated for luminance and are seen against a dark background. Under these conditions there will still be some residual differences in brightness [32]; but these differences among stimuli are relatively small, which makes our stimuli approximately equal in brightness. In any case, all participants viewed the same stimuli, so that brightness differences alone should not account for the sex differences we report here.

We present here data gathered with our scaling techniques from large samples of color-normal participants. We find sex differences in color appearance of monochromatic lights across the entire spectrum.

The sex differences are unexpected, partly because, as we note later, there are large inter-individual differences in cone ratios and cone distributions across the retina [33]. Despite these variations, human color vision is remarkably similar across the population. And yet despite this overall similarity, there are still small, but very real, sex differences. The mechanisms that determine hue and saturation are cortical, meaning that the neuronal inputs from the thalamus have to be rearranged and re-combined (e.g. [30,34]); much of this may take place in primary visual cortex [35]. However, the complete recombination is probably done in several stages: one piece of evidence favoring multiple stages is from an individual who had severe dyschromatopsia (colors were severely washed out and difficult to identify), but without loss of color discrimination [36]; see also [37]. Furthermore, color appearance probably includes several cortical areas beyond the occipital lobe (e.g. [38,39]). Given the sex differences that we are reporting here, this implies that the 23^rd^ pair of chromosomes exerts an impact on this re-arranging of the neuronal pathways from thalamus through several regions of visual cortex.

## Methods

### Participants

All participants were volunteers, drawn from undergraduate and graduate students, and faculty at Brooklyn College, together with some high school students. The demographics of student participants parallel the demographics of the student body at Brooklyn College.

All participants were screened for normal color vision using the familiar plates of figures composed of dots of different colors (Dvorine pseudo-isochromatic plates, Harcourt, Brace & World). The quality of their color vision was assessed with standard panel tests: sets of colored "chips" that had to be arranged in color-order (Farnsworth Dichotomous Test for Color Blindness, Panel D-15, Psychological Corp, and Lanthony’s Desaturated 15 Hue Test, Luneau Ophtalmologie, Paris). All tests were appropriately illuminated by light with a color temperature of approximately 6800 K; viewing distance was 50 cm. For these panel tests, numerical indices were computed to characterize any reversals in the sequences of the colored test caps [26,40]. Each eye was tested separately; eye-sequences were randomized. All participants had normal color vision and all index values were well below the cutoffs established for the panel tests.

There were 58 participants for the Newtonian-viewing condition; 37 females and 21 males (mean age = 24.9, median = 23.0, sd = 9.3, range = 16–61 yrs). For the Maxwellian-viewing condition, there were 47 participants; 32 females and 15 males (mean = 24.2, median = 22.0, sd = 7.8, range = 16–51 yrs). We have found that under our conditions and with our methods, color appearance remains very stable across this age range (see, e.g. [41]).

The studies using these two optical systems (described below) were run at different times and for different purposes. Because there was no overlap in the groups of participants, we first analyzed the data from these two groups separately. From our previous work, we knew that the scaling data would not be the same for each optical system (viewing condition) [25]. As described in detail below, we used a color-difference score to amalgamate the two sets of data.

The study was approved by the Institutional Review Board of Brooklyn College, where all the studies were conducted. All participants were volunteers and gave informed consent to participate in this study. The experiments were conducted in accordance with the principles embodied in the Declaration of Helsinki (Code of Ethics of the World Medical Association).

### Apparatus and procedures

#### Newtonian- and Maxwellian-View optical systems

Stimuli were monochromatic lights, each consisting of a narrow portion of the visible spectrum; these narrow bands were spaced regularly across the spectrum. For both viewing conditions, these monochromatic lights were provided by grating monochromators with triangular exit spectra and half-power bandwidths of 12 nm. Filters were used, where necessary, to block second-order spectra. Illumination was from tungsten-halogen sources. All stimuli were adjusted to be equally luminant under photopic-sensitivity ("daylight") conditions; this was done by adjusting lamp voltage. Stimulus durations were controlled by electromagnetic shutters, placed at focal points of the light sources, and driven by digital timers. For both viewing conditions, a participant’s head was stabilized with a rigid chin and forehead rest, and stimuli were seen against a dark background. For Newtonian-viewing, stimuli appeared on a rear-projection screen. For Maxwellian-viewing, light was focused so that it entered the eye through the central 2 mm of the pupil.

All lights, from these optical systems and from the anomaloscope (see below), were calibrated with a scanning spectro-radiometer/photometer (Photo Research, Model 703A/PC). All wavelength scales (photometer and monochromators) were periodically checked using the emission lines of a Mercury and Argon standard lamp (Oriel). For Maxwellian-viewing, retinal illuminance was derived according to the method of Westheimer [42]. For Newtonian-viewing, the luminance of the screen was measured from the participant’s position and converted to retinal illuminance using Table 15 in Le Grand [43].

Stimuli were circular 1^0^ patches, 500 ms duration, with a minimum inter-trial interval (ITI) of 20 s; this ITI ensured a stable state of adaptation. Stimuli were viewed foveally against a dark background in a darkened room. Retinal illuminances were about 27 Td (retinal illuminance depends on both the area of the eye’s pupil and the luminance of the stimulus). For the Newtonian condition, stimuli ranged from 430 to 660 nm in 10 nm steps. Stimuli were presented once each in random order in a block; the first block was "practice" and not included in analyses; data were averaged across the remaining four blocks; thus, each participant’s data points are means of four repetitions. The only change for the Maxwellian condition was that stimuli ranged from 440 to 660 nm; this was because the amount of light available at short wavelengths differed between the optical systems.

#### Scaling procedures

All five stimulus blocks were presented, one after the other, in a single session. Using either optical system, an individual’s complete set of hue and saturation functions was obtained in one session lasting about 1 hr. At the start of a session the participant dark-adapted for about 10 minutes.

Color appearance of each flash of monochromatic light was described using our form of magnitude estimation procedures of hue and saturation scaling: after each flash, participants stated the percentages of their hue sensations that were R, Y, G, or B, for a total of 100%; multiple names were permitted; they then stated the percentage of the sensation that was chromatic (saturation). Participants responded verbally, and the experimenter immediately entered their responses into a computer. They were told if their hue responses did not sum to 100%, and the trial was repeated – a very rare event.

Participants were not given any specific training in how to use these scales. We have used these procedures on hundreds of individuals, from experimental participants to students in laboratory courses. Most accepted the instructions immediately. Those who complained about difficulty in applying numbers in this way, were simply told to "just do the task." All did the task with equal reliability, as we have previously reported [24,26,27,41].

Our use of bounded percentage scales leads to variances that are greatest in the middle of the range of values and least at each extreme. To normalize the variances associated with data presented here, an arcsine transform was applied to each individual datum prior to any averaging of an individual’s rating of a stimulus [24,44]. Next, each individual’s hue values were re-scaled by their associated saturation values, so that the hue values now summed to the saturation value. This re-scaling is valid, because we have shown that our scaling methods yield ratio scales [24,27].

The re-scaling incorporates the saturation data into the hue curves. Because R and G are largely mutually exclusive (no participant used R and G simultaneously to describe a sensation), as are Y and B, the data can be reduced to a pair of independent spectral functions: R vs. G and Y vs. B; these functions can then be represented on a two-dimensional color space that describes color appearance for the specific set of viewing conditions.

The data were re-plotted on a two dimensional Uniform Appearance Diagram (UAD), whose orthogonal axes are R vs. G and Y vs. B. To represent the results in continuous fashion, the data were fitted with a smooth (cubic) spline [45]. Because we have shown that the UAD for any specific data set has a uniform metric, we can use the spline curve to derive various other color functions. In this paper we use participants’ UADs to find, by interpolation on the fitted splines, the wavelengths needed by each individual to elicit a range of specific hue sensations (e.g., 90%R and 10%Y), as well as the wavelengths of the unique hues, sensations with only one hue component (e.g., B, or G, or Y). We also derived individuals’ wavelength-discrimination functions [23,26,27].

#### Anomaloscope

Almost all of the participants in the scaling studies who used Newtonian-viewing also used an optical system (anomaloscope) in which, in one half-field, the luminances of an additive mix of R-appearing and G-appearing monochromatic lights were adjusted to match the appearance of the other half-field, which was illuminated by a Y-appearing light, whose luminance was kept constant -- the Rayleigh match. Participants were dark-adapted for approximately ten minutes prior to making the Rayleigh match, which took from 10 to 30 minutes, depending on the participant. This procedure was usually done in a single session on a day separate from other tests.

The test field in our anomaloscope subtends slightly less than 2^0^ visual angle; the field, at a distance of 38 cm, is viewed monocularly through a small peephole. Independent, computer-controlled, beams of light emanate from integrating spheres, whose apertures are the test fields. The light sources are high intensity LEDs. Beam-splitters, filters, and computer-controlled stepping-motor-driven field-stops create different full- and half-field stimuli. The appearances of the different beams are: red (R; dominant wavelength = 668 nm, half-power-bandwidth = 28 nm, CIE31x:y = 0.72:0.28), yellow (Y; dominant wavelength = 594 nm, half-power-bandwidth = 14 nm, CIE31x:y = 0.58:0.42), green (G; dominant wavelength = 532 nm, half-power-bandwidth = 16 nm, CIE31x:y = 0.26:0.71); filters (Wratten # 15) limit all spectra to wavelengths longer than about 520 nm. Participants use a numeric keypad for input to a computer that controls the output of D/A channels which, in turn, control linear operational amplifiers that gate high-current transistors driving each set of LEDs.

For the Rayleigh matches, the luminance of the Y standard half-field was always constant (approximately 15 cd/m^2^). The matching half-field was presented at each of 11 starting luminance ratios of RG (0%R to 100%R in 10% steps) in random order. For each one, the participant adjusted the ratio until the additive field looked as similar as possible to the standard; finally, the participant could adjust the overall luminance of the matching field, while the RG ratio was kept constant. If still not satisfied with the match, the participant could go back and readjust the RG ratio and its luminance.

## Results

### Color appearance: hue and saturation scaling

**Figure 1:**
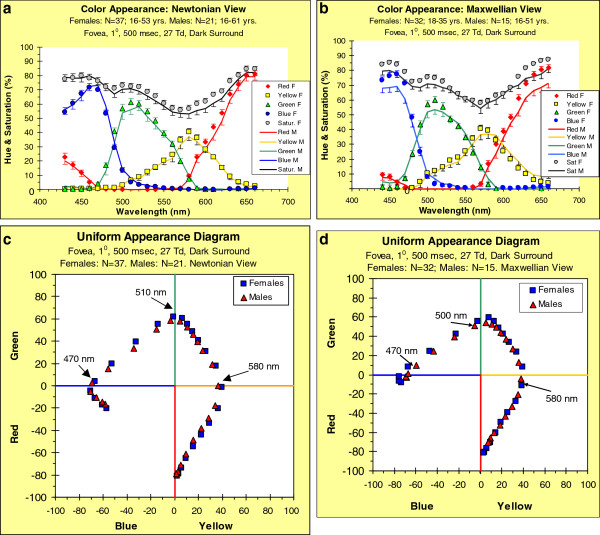
**Color appearance in the fovea of monochromatic lights. Group means of individual hue functions re-scaled by the associated saturation values; hue values sum to the saturation values.** (**a**) Stimuli seen in Newtonian view. Error bars (SEMs) extend above the symbols for females and below the lines for males. (**b**) Maxwellian view. (**c**) Data from Newtonian view smoothed and re-plotted on a two-dimensional color space (Uniform Appearance Diagram; see text for details). (**d**) Data from Maxwellian view smoothed and re-plotted on a Uniform Appearance Diagram.

Figures 1a and 1b show, for the Newtonian-View and Maxwellian-View, respectively, group mean hue scaling data as a function of wavelength. The hue values have been re-scaled by their associated saturation functions such that the hues sum, not to 100%, but to the associated saturation values (see above); the means for females are shown by symbols and the means for males by continuous lines. The error bars are SEMs; for clarity, those for males extend only below the data points, while those for females extend only above the data points. At each wavelength, the group means, and their SEMs, were obtained by averaging the R, Y, G, B values obtained from each participant.

There appear to be sex-related differences, but they seem small, and it is not easy to appreciate their magnitude or direction. The effects are clearer when the data in Figure 1a, b are displayed in a color space, the UAD, in Figure 1c, d. The data for females are rotated slightly with respect to those of males: in most parts of the spectrum, the rotation of the female data is clockwise with respect to the male data – this rotation is implicit in the data and is not the result of an analytic manipulation. Consider, for example, the points labeled 510 nm in Figure 1c; for females, the point is almost on the G-R vertical axis, meaning that the sensation is close to unique G; but for males, the same wavelength is still within the BG quadrant, meaning that its wavelength must increase by a few nanometers in order for it to appear unique G.

We are dealing with data from two viewing conditions. We have previously shown that these conditions result in systematic differences in the hues elicited by each wavelength [25]. We digress to show that the sex-related differences that are the central point of this paper are not due to any differences in viewing conditions.

Although the results were qualitatively similar, there is a problem that prevents us from simply amalgamating the two data sets. Despite having the same retinal illuminance there is an important difference: our stimuli were presented as brief flashes with a minimum ITI of 20 s in a darkened testing room, which meant that participants’ pupils were widely dilated. Thus, in the Newtonian-View much of the light entered through the periphery of the pupil and therefore struck the receptors at angles greater than those for light entering through the pupil center, as is the case in the Maxwellian-View. Such "edge" rays are known to produce changes on color appearance of monochromatic lights (Type-II Stiles-Crawford Effect (SC-II); [46,47]).

**Figure 2:**
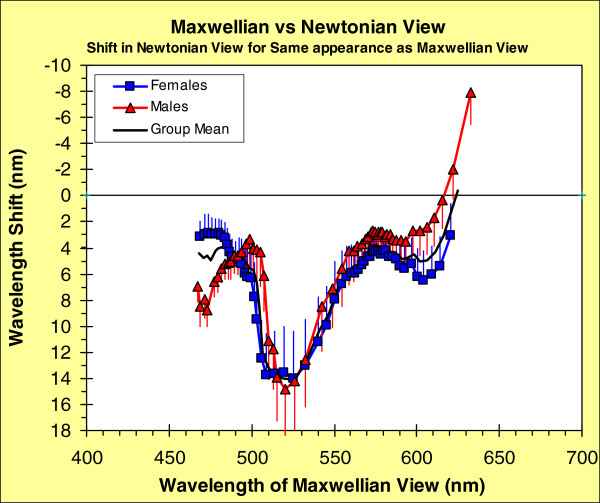
**Shift in stimulus wavelength required to make color appearance seen in Newtonian same as when seen in Maxwellian view.** Group mean functions disaggregated by sex. Error bars (SEMs) extend above the symbols for females and below the symbols for males.

Figure 2 compares our data from the two viewing conditions. The abscissa shows, for Maxwellian-view, the wavelengths that elicited a series of hue sensations, ranging from 100%B to 20%Y & 80%R, in 5% hue steps. (The range of hue ratios is restricted to those that were seen by all participants.) The ordinate shows the change in wavelength needed to produce the same hue sensation in Newtonian-view as from Maxwellian-view. The solid, group-mean, curve is re-drawn from our earlier paper comparing these viewing conditions; possible reasons for the effect are discussed fully in that paper [25]. The other two curves in Figure 2, from the data in this paper (Figure 1c, d), break down the effect by sex: there appear to be some sex-related differences, but they are not significant (see statistical analysis below). 

### Statistical analysis of sex differences

Although the effects of sex on color appearance seemed consistent between the two viewing conditions, they were small and not identical. To demonstrate that the sex effects are real, we used an accepted way to amalgamate data sets that have different means: computation of individual differences from the means for each condition.

Because there were almost twice as many female participants as males, averaging across all participants would grossly bias the result towards the wavelengths required by females for each hue. Therefore, for each viewing condition, a global mean of the wavelengths required to elicit each hue was derived: Optical-System Mean = (Mean for males + mean for females)/2. Means were computed separately for each sex to remove the effects of differences in sample sizes between males and females.

Then, for each hue sensation from one of the two viewing conditions (Newtonian or Maxwellian), each participant’s required wavelength was subtracted from the mean wavelength for that participant’s sex. These differences from each Optical-System Mean, were combined into one large matrix, that was organized to retain sex and optical system descriptors.

**Figure 3:**
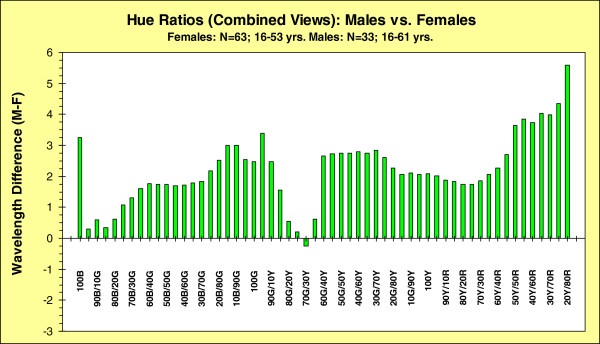
**Sex differences in shifts in wavelengths (combined data from Newtonian and Maxwellian views; see text for description of combination procedure) associated with specific hue sensations.** Wavelengths required for females to experience a specific hue ratio (blue, blue/green, green/yellow, yellow/red) subtracted from the wavelengths required by males for the same hue ratios.

The male–female differences in the wavelength required for a specific hue are shown in Figure 3. This figure shows clearly the central point of this paper: males require a slightly longer wavelength than do females to experience the same hue.

In Figure 3 the abscissa is a series of hue sensations, ranging from 100%B to 20%Y & 80%R, in 5% hue steps; the range of hue ratios is restricted to those that were seen by all participants. (For example, only 30 females reported a sensation of 5%R & 95%B, and only 24 had a sensation of 25%R & 75%B; of the males, only 18 reported a sensation of 5%R & 95%B, and only 14 had a sensation of 25%R & 75%B.) The results from the matrix combining all the data were averaged separately for males and females: the mean wavelength needed to elicit each hue for females was then subtracted from that for males. The results are plotted on the ordinate of Figure 3. For 56 out of 57 of these sensations, covering most of the visible spectrum, males require a longer wavelength than do females to experience a given hue sensation. This difference is also shown in Figure 1c, d: as we have noted (see above), for each viewing condition, the female data are rotated clockwise with respect to the male data.

An ANOVA (SPSS; general linear model, repeated measures, mixed design) was run using the above global matrix: the factors were hue, sex, and optical system. (See Table 1.) While the sex effects were small, the effect of sex was significant: F(1, 92) = 7.004, p = 0.010. The degrees of freedom (92) were slightly less than the degrees of freedom expected from the total number of participants (105); this was due to some missing data points caused by minor errors in the computerized data acquisition. Data from participants with such missing data were excluded from the statistical analysis.

**Table 1:** Analysis of Sex and Color Appearance Between-Subjects Effects

**Source**	**df**	**F**	**p**
SEX	1	7.00	0.01
View (V)	1	0.01	0.93
SEX * V	1	0.50	0.48
Error	92		
Within-Subjects Effects			
**Source**	**df**	**F**	**p**
Hue	56	0.09	1.00
Hue*SEX	56	0.60	0.99
Hue*V	56	0.06	1.00
Hue*SEX*V	56	0.90	0.68
Error	5152		

In Figure 3 the mean effect-size of the male-female differences in wavelength required to elicit each hue is 2.2 nm. Although, across the hues, there are variations in the differences, they are not significantly different from this mean (ANOVA: no significant effect of hue, see Table 1). That is, regardless of the particular hue, males required, on average, a wavelength 2.2 nm longer than the wavelength needed to elicit the same sensation from females. Similarly, there was no significant effect on the difference scores due to optical system: the results were the same when each participant’s data-set was compared to the appropriate mean for a given optical system. Among other things, this means that the possible sex differences in the data from the two viewing condition (see Figure 2) are not statistically significant. There were no other significant effects or interactions.

### Rayleigh anomaloscope matches

Humans are polymorphic for the L- and M-cones (e.g. [13]). Furthermore, individuals may express more than one of these alleles, and there are sex differences in the relative numbers of L- and M-cones [14,48,49]. It is generally agreed that many females have phenotypes with multiple L- and M-photopigments. However there is some consensus that males may express only one of each (e.g. [50]). These findings may be the basis for the significant sex effects on hue that we report here.

Inter-individual variations in spectral sensitivities of L- and M-cones in individuals should affect their Rayleigh anomaloscope matches. In this test a bipartite filed is illuminated on one side with a light that appears Y; the task is to match its appearance with an additive mix on the other side with two lights, one appearing G and the other appearing R. (Because all the wavelengths we used were longer than 520 nm, S-cones contributed essentially nothing to the outcome.) Most participants who scaled the appearances of monochromatic lights seen in Newtonian-view also used our anomaloscope to make Rayleigh matches.

**Figure 4:**
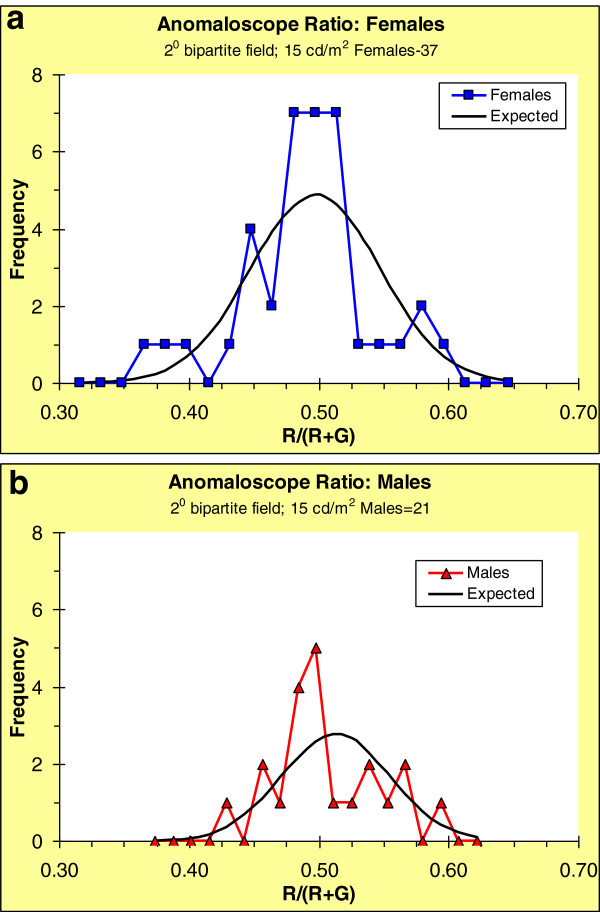
**Frequency distributions of anomaloscope matching ratios: R/(R + G), where R and G are re-weighted to produce a group mean of 0.5 (see text for details).** (Only participants who also scaled hue and saturation of stimuli seen in Newtonian view.) (**a**) females. (**b**) males.

Following the convention of Neitz and Jacobs [48], we derived an average measure of the matching RG value as follows. We pooled the RG ratios for all participants, regardless of sex, and weighted the R and G values so that the group average for the quantity R/(R + G) equaled 0.5. This value is the midpoint of the abscissae in Figures 4a, b; these figures show the frequency distributions of this ratio for females and males respectively. High values of the ratio imply less effective R in the matching mix and low values imply less effective G. Individuals who lack the L-cone will have extremely high values for the ratio, while those who lack the M-cone will have extremely low values; these individuals exhibit a form of color blindness (dichromacy) referred to as protanopia or deuteranopia respectively; moderately high values indicate less severe (anomalous) forms of these deficiencies. All of our observers had ratios far from these extremes – they were color-normal. 

Because humans, particularly females, are polymorphic for the L- and M-cone genes, population Rayleigh matches might be expected to show multiple modes, as was shown in published data based on large samples: males had a bi-modal distribution, while females had a largely tri-modal distribution (e.g., [14]). Even though our distributions of matching RG ratios (Figure 4) do not differ greatly by sex, they do show, especially for the females, some of the sex-related differences reported previously. Each graph also includes the normal distributions expected (based on group means and variances) if only random variations were involved. Applying the Kolmogorov-Smirnov test (as included in SPSS), the frequency distribution for males was not significantly different from normality (p = 0.134), perhaps due to small sample size; for females there was a significant difference from normality (p = 0.016).

We considered the distributions of Rayleigh matches because significant multiple modes point to possible sub-populations; each sub-population could be associated with multiple modes in spectral distributions of unique hues. We examine this below.

### Unique hues

Single cones can only report the rate at which their photopigments are absorbing photons – once a photon is absorbed, all information about its wavelength is lost. To provide information about wavelength (color), the nervous system must compare the responses of cones that contain different photopigments; this comparison is done by spectrally-opponent cells in the retina – for example, a cone type that is more sensitive to longer wavelengths might excite these cells, while another cone type, more sensitive to shorter wavelengths, would inhibit them.

Spectrally-opponent systems seem ubiquitous in species with color vision, ranging from assorted shallow-water mullets of the family Mugilidae [51], to eels [52], to macaque monkeys [53]. In the macaque, four types of spectrally-opponent cells have been identified in the retina and visual area of the thalamus (lateral geniculate nucleus) [53-56]. These opponent cells have spectral points at which excitation and inhibition are equal and there is no net response (null point). Psychological sensations of color also have spectral nulls – for example, the sensation that is only Y (unique Y) coincides with the null point for R vs. G (see Figure 1). However, the psychophysical null points (unique hues) do not coincide with the nulls of the spectrally-opponent cells. Sensations must ultimately depend on re-processing of these neural inputs to determine opponent hue (sensory) mechanisms [30].

The spectral loci of the unique hues are especially interesting because they define the null points of spectrally-opponent sensations – i.e., hue mechanisms. We argue that hue mechanisms are opponent, based on a variety of evidence, including observations that one half of an opponent system can be used to cancel the sensation of its opponent [57,58]. Thus, unique Y occurs at the wavelength that elicits a sensation of neither R nor G; this is the null point of the RG mechanism. The precise values of these loci therefore play an important role in constraining many models of color vision based on spectrally opponent processing of visual information (e.g., [30,57,59-61]).

**Figure 5:**
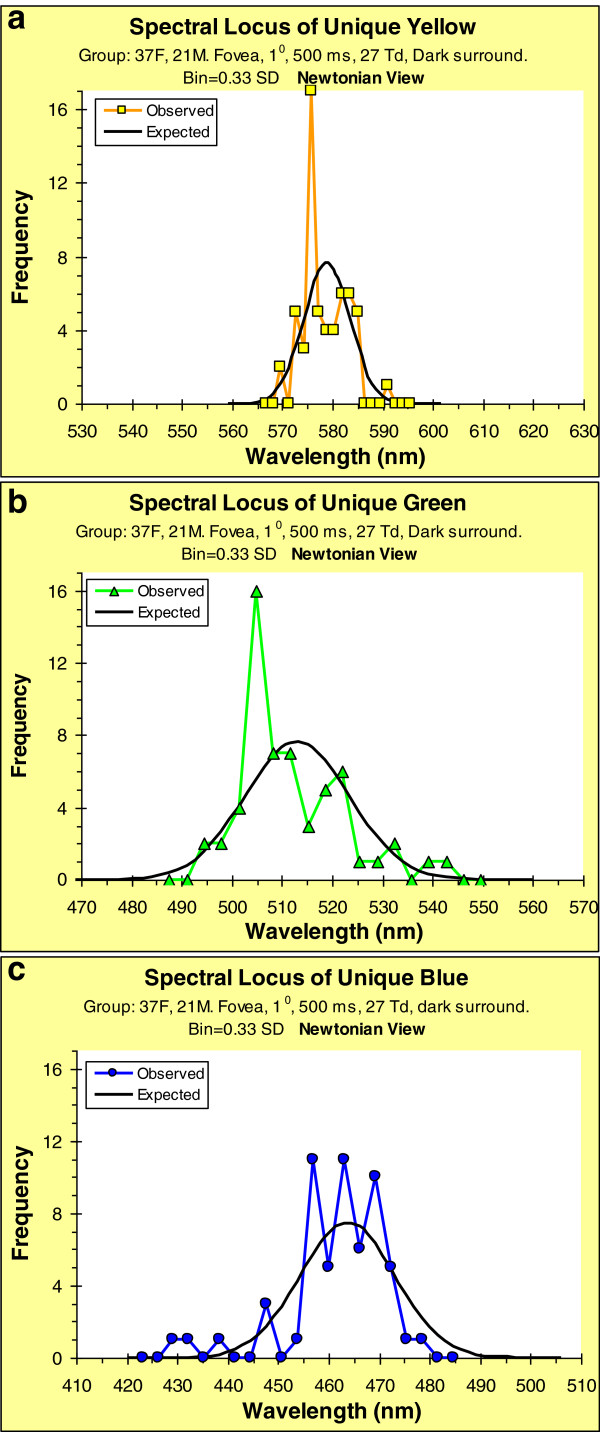
**Frequency distributions of spectral loci of unique hues (Newtonian view), together with normal distributions based on means and standard deviations of the data.** Bin widths = 0.33 of SD for given distribution. (**a**) Yellow. (**b**) Green. (**c**) Blue.

UADs were plotted for each individual; the wavelength for each unique hue was found by interpolation on the fitted spline. Figures 5a–c show the frequency distributions of the spectral loci of unique Y, G, and B. In these figures, for simplicity, we show only data for the Newtonian-view – the results and conclusions for the Maxwellian-view are very similar (e.g., see Table 2). For comparability across these three graphs, bin widths were set at 0.33 of the standard deviation for each distribution. (Some of the data points in these figures were included in [41], but here we have added a substantial number of new participants.) Note that for most individuals there is no spectral wavelength that corresponds to unique R – the longest wavelengths elicit a sensation that contains some Y. For each hue, we also show the expected distribution if the loci were normally distributed. From Kolmogorov-Smirnov tests (as included in SPSS), all the group data distributions differ significantly from their expected normal distributions: for Y, p = 0.0043; for G, p = 0.0004; for B, p = 0.0003. The significant differences from normality and the existence of sub-peaks suggest that for the unique hues, humans are not a homogeneous population. In particular the distribution of Y is very narrow, a finding that has also been reported by others using comparable sample sizes but very different psychophysical techniques [62]. 

**Table 2:** Spectral Loci (nm) of Unique Hues for Newtonian and Maxwellian Views

**Newtonian View**				**Maxwellian View**			
	Y-unique	G-unique	B-unique		Y-unique	G-unique	B-unique
Males	579.9	513.5	466.5	Males	577.1	507.4	463.2
Females	578.4	512.8	462.3	Females	574.2	503.3	461.7

**Figure 6:**
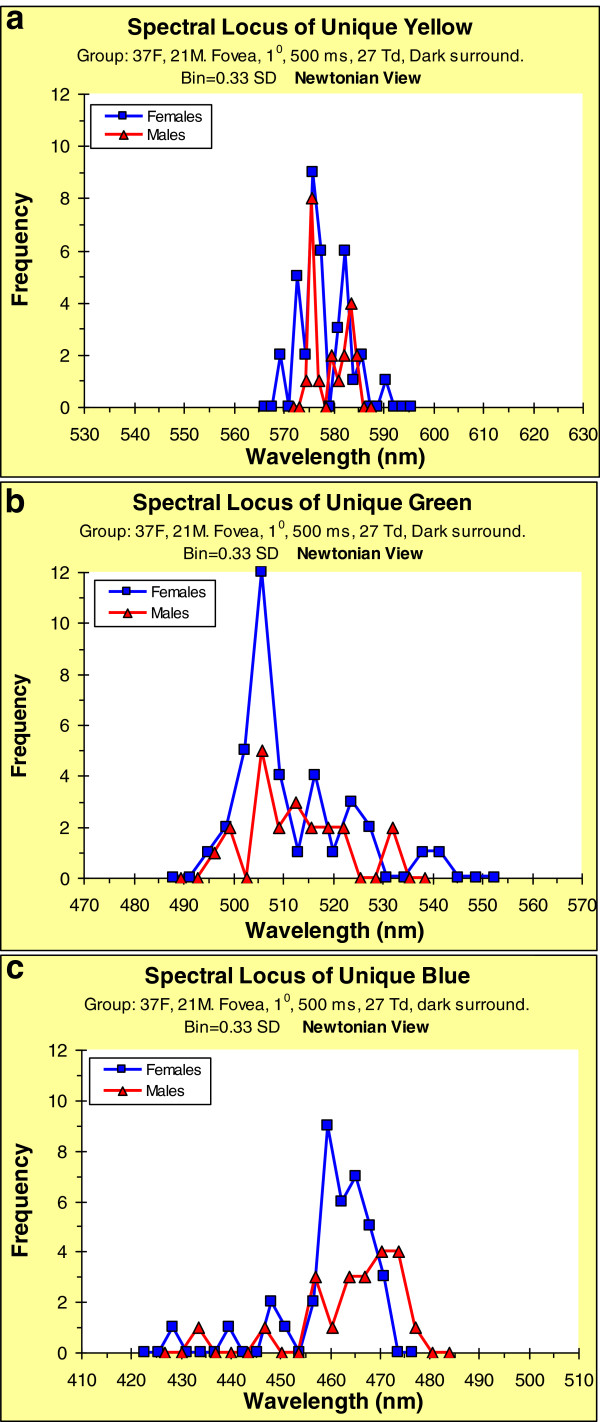
**Frequency distributions of spectral loci of unique hues (Newtonian view), disaggregated by sex. Bin widths = 0.33 of SD for given distribution.** (**a**) Yellow. (**b**) Green. (**c**) Blue.

The multiple peaks seen in the distributions in Figure 5 may be sex-related; Figures 6a–c show the same data, but split between males and females to examine this. Applying the Kolmogorov-Smirnov test, the frequency distributions of females show significant deviations from normality: Y, p = 0.01; G, p = 0.0005; B, p = 0.0002. However, none of the male distributions differ significantly from expected normal distributions (possibly due to small sample size): Y, p = 0.152; G, p = 0.2; B, p = 0.119. But in all these cases, males have their loci shifted towards longer wavelengths, which reiterates the general finding that males require a longer wavelength than females to experience the same hue sensation (Figure 3).

The similarity of the findings for the two viewing conditions is shown in Table 2: Regarding the spectral loci of the unique hues, the group mean wavelengths, for Newtonian and Maxwellian views, are given in Table 2. Not only are the values similar, but in all cases the values for males are shifted to longer wavelengths.

To examine whether there were any correlations among individuals’ spectral loci and their associated anomaloscope ratios, we computed R^2^ values separately for males and females. None of the correlations, for any of the unique hues, was significant; most were essentially flat lines with R^2^ ranging from 0.001 (males, B-unique) to 0.17 (males, Y-unique). The lack of any clear correlations is interesting. Given the relatively broad range of the anomaloscope ratios, and the indications of sub-peaks, possibly related to expression of different L- and M-cone alleles, we might have expected a closer relation between a participant’s anomaloscope ratio and his or her locus of a spectral hue. Unique Y in particular is a function only of L- and M-cone inputs to the G-R opponent hue mechanism; it coincides with the spectral null point of the G-R system. But, these cone inputs must be weighted relative to each other: specifically, the input from the M-cones must be weighted more strongly than that from the L-cones in order to shift unique Y to its observed spectral locus.

Furthermore, the relative weights of these inputs must be quite tightly constrained because the distribution of unique Y shown in Figure 5 is narrow (see [30] for a more complete discussion). This narrowness is remarkable for two reasons: firstly, there are differences in sensitivity among L- and M-cone spectral sensitivities, as shown by the range of anomaloscope ratios; secondly, there are large variations among individuals in the relative numbers of these cones [33]. The cortical weighting of the cone inputs to the G-R system must compensate for these individual differences.

Finally, we looked for any possible correlations between the spectral loci of each pair of unique hues. We found none, confirming similar earlier conclusions [61,63].

### Wavelength discrimination

Because an individual’s UAD for a particular viewing condition has a uniform metric, it can be used to derive a wavelength- discrimination function [23-27]. Participants’ functions were derived by measuring, for each stimulus, (along the spline function fitted to each individual's UAD – see above) the change in wavelength needed to produce a fixed, criterion change in sensation; these wavelength shifts were averaged across participants to obtain group wavelength-discrimination functions for males and females.

**Figure 7:**
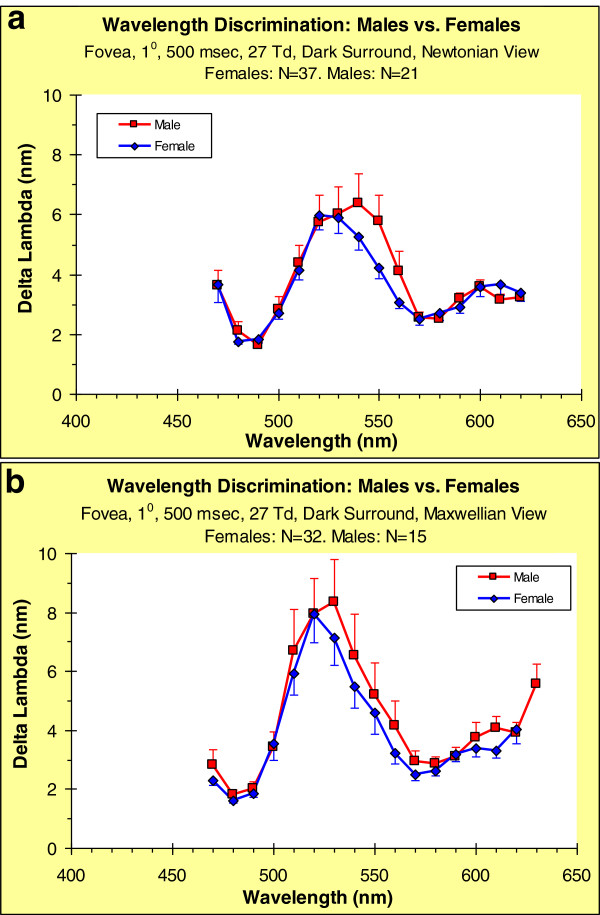
**Group means of wavelength-discrimination functions derived from individual Uniform Appearance Diagrams; disaggregated by sex.** (**a**) Newtonian view. (**b**) Maxwellian view. Error bars: SEMs.

Figure 7 shows wavelength-discrimination functions for the two optical viewing conditions broken down by sex. In Figure 7a we show the curves for the Newtonian View, and in Figure 7b the same for the Maxwellian View. The general trends are remarkably similar. While there are no statistically significant sex differences, the male and female curves are not identical. Applying Exploratory Data Analysis [64] to these data: there appear to be systematic differences between the sexes. In the middle of the spectrum, males have a slightly broader range of relatively poor discrimination (540–560 nm for Newtonian-view; 530–570 nm for Maxwellian-view). We suggest that the sex differences in wavelength discrimination are real. 

## Discussion

We have shown that there are significant differences between males and females in the appearance of monochromatic lights. The color-appearance spaces that we have derived are similar between the sexes, but they are not congruent – one is rotated with respect to the other. Across most of the visible spectrum males require a slightly longer wavelength than do females in order to experience the same hue. What might be the factors behind these differences?

Human color vision is trichromatic. Historically it had been assumed that this trichromacy was based on our having three spectrally distinct cone types: color matches occurred when the simultaneous photon capture rates of each of the cones types from an additive mix of three primary lights matched the capture rates from the light being matched (e.g., [47,58]). However, it is now clear that this is too simple: humans have multiple alleles for the opsins that form the L- and M-cone photopigments (e.g., [13]). Markers for these variant opsins show that many humans, especially females, express more than one of the variants in their phenotypes [14,48,49].

Despite the fact that many humans have more than three cone photopigments, all behave as if they have only three primary channels. They must have S-cones plus some unitary form of each of an L- and M-cone: all can make a Rayleigh match on an anomaloscope, in which a Y-appearing field is matched by an additive mix of only two primaries, one that appears R, and one G. (To match all possible lights, a third B-appearing primary would be needed, e.g., [65].) All color-normal phenotypes make Rayleigh matches with the same precision and with closely comparable ratios of the primaries in the mix, although there may be small systematic differences in the ratios that depend on the precise pigments expressed (e.g., [14,48]). Despite the many careful studies, with large samples of participants, that point to human trichromacy, there are still claims that some females who express multiple L- and M-cone alleles are tetrachromatic [66]. However, a recent, large and precise study of these issues found only one heterozygous female carrier of deuteranomaly who appeared to be tetrachromatic; this individual could not make a Rayleigh match on a standard anomaloscope [67].

Trichromacy implies that if a person expresses, for example, two versions of the L-pigment, the responses of these cones must somehow be combined by the nervous system to function as a single L-channel. We have argued [30] that this most probably occurs not at the level of the retina, but at the cortex, for the following reason: in the fovea and immediate peri-fovea, the centers of the receptive fields of retinal ganglion cells are driven by single cone types [68,69]; for example, some ganglion cells might have L1 centers and some L2 centers, which would also be true of the LGN, because there is a one-to-one connectivity. The first level for possible combination into a single L-channel is the cortex. We will return to this when we consider the implications of the sex differences we report.

It is now commonly accepted, at least by those whose prejudices about color vision accord with ours, that hue mechanisms are based on two spectrally-opponent mechanisms: R vs G and Y vs. B [22,70]. Spectrally-opponent neurons at the levels of retinal ganglion cells and LGN cells can be subdivided into four sub-types [53,54]. But these cells cannot be hue channels, because their spectral responses do not correspond precisely to individuals’ sensory responses. The responses of spectrally-opponent neurons are aligned along specific directions in cone-spectrum space (often referred to as DKL axes) [65]. This space must be rotated to coincide with hue (sensory) space. The rotation must be achieved by re-weighting and re-combining LGN spectrally-opponent inputs to the visual areas of the cortex (e.g., [30,34]). It can be shown [30] that it is possible to re-combine LGN responses so as to produce two spectrally-opponent mechanisms, from which one can derive in detail hue, saturation, and luminosity functions closely similar to those from psychophysics.

At this stage it is unclear how and where the relevant neuronal information corresponding to psychophysics is extracted. Certainly, no single cells have been recorded whose responses correspond directly to hue mechanisms. It is possible that the sensory functions we have dealt with represent responses of ensembles of cells that are distributed across several cortical levels. For example, damage to a specific region of infero-temporal cortex, the fusiform gyrus, produces achromatopsia, the inability to describe hue sensations [71], but may not interfere with wavelength discrimination [36].

The problem with all this seemingly necessary re-organization at the cortical level is that it must differ greatly from individual to individual: for example, there are large differences in the LM cone ratios among individuals, and yet their color vision seems remarkably similar [72]; an example of this similarity is the tight distribution of the spectral loci of unique Y that we and others have observed (see Figure 5a). There are even greater variations across any individual’s retina, with the periphery being overwhelmingly dominated by L-cones [73]; despite this, the spectral loci of the unique hues remain stable across the retina, provided peripheral stimuli are made larger [41,74-76]. This means that the re-organization must also vary with retinal eccentricity.

In the face of all these variations, how does the cortical re-organization of the LGN inputs iterate to essentially the same points across individuals? We know that even in adults the system is plastic and will respond to gross changes in the environment (such as wearing colored goggles), which will shift the loci of unique hues [77]. But what is the tuning signal, under normal circumstances, that changes the relative weights of the different cone types across individuals and across a single individual’s retina? A common suggestion is the "gray-world" hypothesis [78,79]: the space average of most real scenes is achromatic, which could provide an external standard. This, however, may be too simple: color appearance must remain reasonably stable over long time periods in the face of changes in average spectral distribution. For example, these changes could include seasonal changes in the environment -- variations in color of foliage and ground cover [80], or changes in the available spectrum due to yellowing of the eye’s lens with age [81].

Thus, hue mechanisms would have to be tuned until they no longer responded to large-scale averages of real scenes, otherwise they would be signaling a specific hue. (And, this would have to be going on while the same mechanisms were signaling the hues of specific objects of interest.) Presumably this re-tuning would also have to be relatively rapid, because the color-appearance of objects does not change grossly when the illuminant is changed (e.g., [82]); this is sometimes referred to as "discounting the illuminant." There are additional problems with the gray-world hypothesis and its variants: careful recordings of real-world scenes show that they vary substantially and so would make rather poor standards for tuning the visual system. However, some sort of long-term adaptation is necessary if objects are to maintain their appearance.

## Conclusions

We have spent much of the discussion on how cone responses lead to color sensations. We have done so to set the context for our findings. In this paper we have added an additional, non-trivial, factor in the neuronal organization of LGN responses that must take place at cortical levels, a factor that is probably distributed across several levels of visual cortex. The initial wiring of the neuronal connections is presumably genetically determined and guided. But other factors modify the underlying pattern to ensure that color sensations across humans are very much the same. We assume that these factors are in some fashion maturational and/or environmental.

We add that the individual’s sex must be included in the mix. The final rotation of the LGN color space to match the sensory space is not the same for the sexes. And, incidentally, this sex difference would seem to argue against some forms of a gray-world hypothesis – the scenes are presumably equally gray for females and males and yet there is a difference in the tuning of their sensory systems. At this point we have no idea how sex provides this influence. In the absence of any additional data we feel it is premature to provide detailed speculations about possible factors.

We note, however, that in the auditory system sex-linked differences are linked to levels of testosterone [4]. Given this finding in a major sensory system and the existence of androgen receptors at the various levels of the visual cortex [8], it seems reasonable to postulate that something similar applies to the visual system. While it is true that the Y-chromosome carries very few genes, it does have the gene for testosterone and may therefore be the basis of the sex effects we report here. However, it is also possible that the effects may be due to some other sex-linked gene, and not necessarily one on the Y-chromosome. Regardless of the locus that controls the effects, it seems obvious that any factor that influences a wide scale re-organization is important for a complete understanding of how color vision in the central nervous system develops into its mature form.

## Competing interest

None of the authors have any competing interests.

## Authors’ contribution

All authors participated in all aspects of this paper: design, testing participants, data collection and analysis, manuscript preparation. All authors read and approved this final version of the manuscript.
